# Path to success: female leaders in German neurosurgery

**DOI:** 10.1007/s10143-023-02163-5

**Published:** 2023-10-14

**Authors:** Miriam Weiss, Rabia Dogan, Ulrike Eisenberg, Aruni Velalakan, Jutta Krüger, Ina Moritz, Dorothea Nistor-Gallo, Charlotte Flueh, Claudia Janz, Rezvan Ahmadi, Karlijn Hakvoort, Marie-Thérèse Forster, “Women in Neurosurgery –Open for all” –Official Commission of the German Society of Neurosurgery (DGNC)

**Affiliations:** 1 Department of Neurosurgery, Cantonal Hospital Aarau, Aarau, Switzerland; 2 Department of Neurosurgery, RWTH Aachen University, Aachen, Germany; 3 Berlin, Germany; 4 Department of Neurosurgery, Ludwigsburg Hospital, Ludwigsburg, Germany; 5 Hamburg, Germany; 6 Department of Neurosurgery, MSB Medical School Berlin, Helios Klinikum Buch, Berlin, Germany; 7 Department of Neurosurgery, University Hospital Erlangen, Erlangen, Germany; 8 Department of Neurosurgery, University Medical Center Schleswig-Holstein, Kiel, Germany; 9 Department of Neurosurgery, Solingen Municipal Hospital, Solingen, Germany; 10 Department of Neurosurgery, University Hospital Heidelberg, Heidelberg, Germany; 11 Department of Neurosurgery, Goethe University, Schleusenweg 2-16, 60528 Frankfurt Am Main, Germany

**Keywords:** Neurosurgery, Women, Gender, Diversity, Leadership

## Abstract

Despite advances in gender equality, only 6% of German neurosurgical departments are currently led by women. With regard to their pioneering work and the importance of their role model effect, we aimed at reporting on the career pathways of the present and former female chairs of neurosurgical departments in Germany. We approached current and former female chairs in German neurosurgery and gathered descriptive information on their ways into leadership positions through structured interviews. Data were obtained from 16/22 (72.7%) female neurosurgical chairs, aged between 44 and 82 years. They completed their training within 6.5 ± 0.6 years, and it took them further 14.5 ± 5.9 years between training completion and chair acquisition. Having obtained their chair positions between 1993 and 2020, six (37.5%) of them have retired or changed career tracks. Of ten (62.5%) chairs still practicing, two are directors of university departments. Twelve (75.0%) hold professorships. Nine chairs (56.3%) are married, eight (50.0%) having children. Five chairs reported having experienced gender-based discrimination. Twelve had a male mentor or role model, two had a female role model, while only one had a female mentor. This study characterizes the to date small number of female neurosurgical chairs in Germany and their paths to neurosurgical leadership positions. In future, these should become historical in order to perceive the presence of women in leadership positions as self-evident normality, reflecting our society. However, further analyses comparing paths of both female and male neurosurgical chairs are necessary to explore gender-based differences in achieving neurosurgical leadership positions.

## Introduction

Awareness for gender (in)equality has been raised in a range of areas in society throughout the past decade. Equal opportunities in the labor market together with equal societal reception of women not only working but holding leadership positions are necessary to reach equality of gender. Medicine in Germany is no exception to a historically higher proportion of male physicians in all positions [1]. In early career stages, this ratio has already changed significantly, with slightly more women than men attending German medical schools today [2]. Since women tend to achieve better grades in secondary schools, this gives them better chances at being accepted into one of the competitive German medical school programs [3]. However, this initial preponderance of women quickly becomes smaller with increasing level of training and rank [4, 5]. In surgical disciplines, including neurosurgery, the so-called “glass ceiling” seems to be particularly pronounced [6]. Only recently, a study identified women making up 35% of residents and 23% of senior physicians in German neurosurgery [7]. While those numbers show that the initial preponderance of women in medical school decreases during residency, the gender imbalance is more striking in neurosurgical leadership positions in Germany (department deputy directors, chief or managing senior physicians), where women only account for 9%. Accordingly, only 6% of all German neurosurgical departments (and 5% of German University neurosurgery departments) are currently chaired by women [7, 8].

The paucity of neurosurgical mentorship dedicated to women and a lack of visible female role models are presumably some important reasons for this persisting gender gap. Collective information on the present and former female neurosurgical department chairs in Germany is still lacking. As an initiative of the official commission of the German Society of Neurosurgery (Deutsche Gesellschaft für Neurochirurgie, DGNC) for “Women in neurosurgery – Open for all”, we characterized the careers of current and former female heads of German neurosurgical departments. Our aims were to analyze their pathway to typical career milestones, identify if and which obstacles were encountered along their way, and increase the visibility of successful neurosurgical female leaders.

## Materials and methods

### Study population

This is a cohort study on current and former female neurosurgery department chairs in Germany by the DGNC commission “Women in neurosurgery – Open for all.” A list of neurosurgical departments in Germany was retrieved from the DGNC and departments with female heads were identified via departmental websites. Additionally, female department heads were searched through a call for participation in the official journal of the German Medical Association (Deutsches Ärzteblatt), by personal knowledge and internet research. Both active and retired female neurosurgery department chairs were included. Gender forms outside the binary allocation were not considered separately.

### Data collection

All identified women were approached for taking part in an interview. Interviews took place in person or via teleconference between 03/2021 and 07/2022. A pre-specified interview structure was used including questions on background information, medical school, residency, senior positions, chairing position, scientific engagement, career obstacles, idols, and private life. The prespecified information was gathered as far as consented by the participants. Each investigator was responsible for one to four interviews. Results were documented both numerically and in a short biography (3–10 pages) each, highlighting noteworthy events, persons or circumstances that had particular impact on career development. Publication counts (first or last authorships, co-authorships) before and after becoming department head were provided by the participating women or were identified by search in Medline and Google Scholar. Consent for publication of anonymized data or, in selected cases by name, was granted by all participating women.

### Career system in German neurosurgery

Neurosurgical residents in Germany are hired after graduation throughout the year whenever a position becomes available. The decision to hire an applicant is made by the department chair. The standard duration of neurosurgery residency training is 6 years. After board certification, neurosurgeons can be promoted by the department chair into senior physician positions, and, after several years as a senior physician, to the rank of leading or managing senior physician or deputy department chair. Applications for department chair positions are usually tied to longstanding clinical experience and substantial scientific contributions. Calls for chair positions are solicited publicly and may optionally include preferences for female applicants as part of a hospital’s or university’s gender equality target. Amongst other requirements, applicants are expected to present a concept for the future management of the department to an application committee from the local hospital or university. The highest-ranked of the short-listed candidates is offered the position after interviews are completed.

### Statistical analysis

Both numeric and descriptive data were gathered. Metric data are presented as mean ± standard deviation, discrete data as n (%). IBM SPSS Statistics 26 (SPSS Inc., Chicago, IL, USA) was used for calculations as appropriate. The graphical elements were created with Microsoft PowerPoint 16 (Microsoft Corporation, Redmond, WA, USA).

## Results

**Fig. 1:**
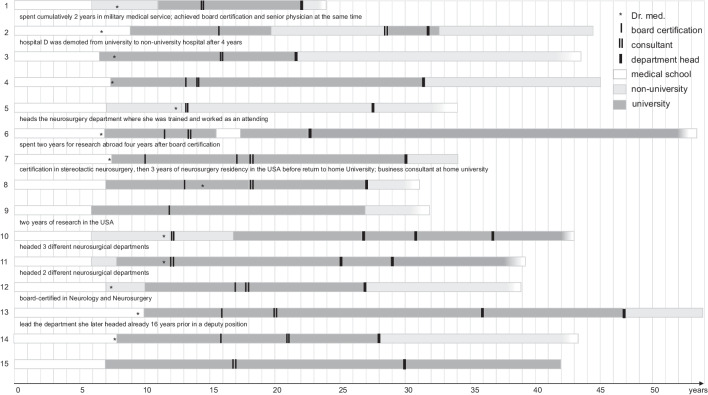
Career timeline of female department chairs in Germany neurosurgery. White blocks indicate time for medical school. Blurry edge on the last block indicates that the female chair is still active, solid edge of last block indicates she is retired. If the woman was promoted into a consultant position at the same time as board certification, two solid lines for becoming a consultant are shown

**Fig. 2:**
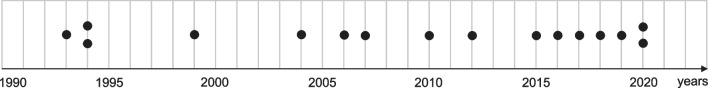
Timeline of women assuming a neurosurgical chair position. Four chairing positions were assumed by women before 2000. After 2000, a chair was assumed by a female about once every two years. The case number is too small to determine if the number of women in these positions is collectively increasing

A total of 22 women chairing neurosurgical departments in Germany were identified, of whom 16 (72.7%) consented to take part in an interview and to be included into this study. At the time of data collection, female chairs were 62.4 ± 9.8 years of age. All but one chair enrolled into medical school in the same year or up to one year after finishing graduate school (Fig. 1). Medical school was completed in standard time (6 years) by nine chairs (56.3%), or with one (*n* = 6, 37.5%) or two (*n* = 1, 6.3%) additional years. All chairs completed a doctoral thesis (German Doctor medicinae, Dr. med.) within 1.8 ± 2.7 years after graduation. They underwent neurosurgery residency training during 6.8 ± 1.4 years, with seven (43.8%) chairs completing residency in standard time (6 years). Seven (43.8%) chairs spent time abroad for clinical training or research during or after residency. One chair completed both neurology and neurosurgery residencies in 10 years, another underwent 3 years of dedicated stereotactic and functional neurosurgery training during residency. The majority of female chairs (*n* = 11, 68.8%) did not change institutions during residency. Fourteen (87.5%) were promoted into senior physician positions within two years after board certification, while it took two chairs three and five years, respectively, to achieve this position. Seven (43.8%) chairs were promoted to leading senior positions before applying for chair positions. Professorships were appointed to 12 (75.0%) chairs 12.6 ± 4.5 years after board certification. Before chairing a department, they published 14.1 ± 16.8 articles as first or last author and 17.9 ± 18.8 articles as co-author. Time from board certification to taking over a department chair position was 14.5 ± 5.9 years. Chair positions were assumed between 1993 and 2020 (Fig. 2), when these female chairs were 48.8 ± 6.1 years old. Ten (62.5%) female chairs are currently holding their position; two are heading neurosurgery departments of university hospitals of which one is a department for stereotactic and functional neurosurgery; eight are heading neurosurgery departments in non-university hospitals, of which one is a department for pediatric neurosurgery. One former chair has changed into a university consultant position and five (31.3%) are retired. Of these, two had changed from a university to a non-university hospital as chairs. Regarding their scientific activity, ten (62.5%) female chairs have continued and substantially expanded their scientific standing (> 20 additional publications and leading a research group) since heading a neurosurgical department.

Partial information was given on private life and career obstacles. While nine female neurosurgical chairs are married, six are unmarried or divorced. Eight chairs have children. Three of those without children deliberately decided against having children in order not to risk compromising their working lives. Three chairs having one or more children reported difficulties combining motherhood with their career ambitions (e.g., finding a daycare). Having had a female role model during her career was reported by two female neurosurgical chairs. Only one female neurosurgical chair experienced female mentorship. Twelve chairs had support from one or more male mentor(s) along their career. Six female neurosurgical chairs did not recall any gender-related obstacles to their career, five others reported having made discriminating experiences based on their gender during their professional lives.

## Discussion

In 1993, Gabriele Schackert became the first female chair of an academic department of Neurosurgery in Germany. As she recalled later, until the position was advertised and she was recommended as a candidate, she had not considered holding such an appointment before, despite her excellent qualifications. At that time, women did simply not hold such positions. Today, almost 30 years later, as Professor Schackert has just retired from her position, we cannot yet observe a clear trend towards increasing numbers of women as neurosurgical department chairs, as in medicine in general [5, 8, 9]. In line with general surgery and urology, the field of neurosurgery holds the lowest proportion of female chairs of all medical disciplines (5% each), despite 31% females in neurosurgical attending positions [8]. The highest proportions of female department chairs in Germany are found in psychiatry (22%), pediatrics (22%) and obstetrics and gynecology (19%), still contrary to the much higher proportion of women in medical schools and residencies. Germany is no outlier in comparison to other countries. In the USA, only one woman holds a neurosurgical department chair despite 216 practicing female neurosurgeons [9]. In Asia and Australasia, there have been 33 women in neurosurgical leadership positions in departments, divisions or faculties of neurosurgical institutions to this day [10]. In the Latin American continent, 16 women have been listed up to now who have served as a neurosurgical department chair. [11]. A multitude of studies exists having explored the reasons for female underrepresentation in the various professional fields, as the trend is certainly not limited to neurosurgery or medicine in general [12–15]. Thus, we were particularly interested in determining whether female neurosurgery chairs in Germany have equally faced such obstacles.

About half of the 16 interviewed female neurosurgical chairs reported gender-based discrimination by superiors during their career. Some alarming observations were reported; for instance, that male colleagues were preferred to assist in surgeries during residency with the claim that women were too weak to perform neurosurgical operations. About 30 years ago, an application for a senior consultant position was denied to one female neurosurgeon without any opportunity for an interview, as it would have been inappropriate to hire a woman in such a high position. In the past, female neurosurgeons were not addressed in speeches or invitations, and even official forms such as the neurosurgery specialist certificate were only issued in the male gender form. Although these experiences are anecdotal and were in part made decades ago, it is unlikely they were and still are complete exceptions. It might be ill-advised to deduce a general trend of discrimination against females in German neurosurgery from a small number of interviews. However, large surveys could also not rule out discrimination against women in surgical disciplines either [7, 16–20]. The rate of “microaggressions” directed against women has been found to be significantly higher than those against men (80% vs. 36%), and stemming predominantly from senior male residents and attendings and male patients [12]. While those interviewed women who made such experiences have become neurosurgical chairs nevertheless, it is the goal of our commission to expose fewer aspiring women on their way to neurosurgical leadership positions to subtle discrimination in the future.

Several interviewed female chairs perceived pregnancy and childcare as one of the major obstacles to a woman’s academic career. While a third of the interviewed female chairs have one or more children, three others actively decided against having children to focus on their career. In 2018, a third of questioned female general surgery residents in the USA would discourage female medical students from a surgical career, specifically based on the difficulties of balancing motherhood with it [21]. Female surgeons may face more work-home conflict and are less likely to get married [13]. A survey among 1118 European neurosurgeons showed that 54% of male neurosurgeons, but only 22% of female neurosurgeons have children [22]. Pregnancy complications may occur more often in female surgeons [23–25], but robust evidence in this regard is missing, as are evidence-based guidelines for operating during pregnancy. By legislature, performing surgery used to be prohibited during pregnancy in Germany until 2018 [26, 27], potentially resulting in a prolongation of residency, lack of surgical training and overall career setback. Since revision of the law in 2018, women are allowed – but certainly not obligated – to operate during pregnancy under particular circumstances and after precautions have been discussed with a local protection officer. To what extent female surgeons in Germany now have a say in their participation in the operating room during pregnancy is object of an ongoing survey. Respective data would be highly interesting for the advancement of initiatives tackling family-career conflicts. Organizing the demanding neurosurgery training and childcare for the following years constitutes another challenge. The German social system has been offering a relatively long, paid parental leave (up to 14 months) since 2007 and every child past the age of 1 year is entitled to a daycare placement [28, 29]. However, the German system still fails to offer sufficient structural support to families with children such that both parents may maintain a full career. In reality, many parents struggle to find an open placement at a daycare and opening hours are not adjusted to surgical working hours [30, 31]. A recent political initiative in the federal government considers cutting the funding for paid parental leave above a certain income [32]. This would likely include some academic, double-income couples such as neurosurgeons, showing that the political priorities to support gender equality, of course, also have financial limitations. While this may appear to be a gender-independent problem, studies show that primarily mothers reduce working hours—voluntarily or involuntarily [33, 34]. The complexity of this circumstance prevents any easy solution and will inherently keep requiring creativity and perseverance during these challenging years. Highlighting the careers of female department chairs with or without children may showcase the different ways this time of life was handled, and the possibility that having children and a successful neurosurgical career do not have to be exclusive.

Whether women do not strive as much as men towards a leadership position or are not promoted there despite equal ambitions is the center of many discussions revolving around female to male ratios in many professions. It is unknown what proportion of German neurosurgery residents strive towards leadership. A survey among German medical residents revealed that only 9.8% of women versus 28.4% of men aimed for a department chair position [14]. Female neurosurgeons were found to exhibit higher levels of self-doubt, anxiety, and impostor syndrome than men, even after having achieved successful leadership positions [35], and leadership is still often associated with a male figure [36]. Hypothetical biological differences in behavior can hardly be separated from—potentially changeable—longstanding traditional gender-related roles that both men and women grew up with in Germany. Of the interviewed female department chairs in this study, only two had a female role model, one had a female mentor and most had a male mentor. Many positives (and negatives) that can be learned may not differ much between a male or female role model or mentor, and the overall symbiosis of that relationship may be much more important than gender. Nevertheless, some data indicate that female medical students may choose a career in surgery more likely in faculties with higher proportions of women [15]. Already when thinking about the neurosurgical specialty, female medical students are more interested in how the specialty will be compatible with having a family [37]. The simple visibility of female leaders with children in local departments might unconsciously resolve such uncertainties..

The commission “Women in Neurosurgery – Open for all” of the German Society of Neurosurgery (DGNC) aims to promote female neurosurgical leaders. Strategies from other fields will be evaluated and may be finetuned for German neurosurgery [38]. These should include incentives on various levels, including [38]: Leadership commitment: to set gender-equity oriented guidelines for DGNC-associated neurosurgery departments; Gender-bias elimination: to analyze and target factors that contribute to gender bias, highlight the normality of women in leadership positions and to reduce implicit reservations against a neurosurgical career and targeting a higher position; Leadership training: to address leadership development already in early career stages, by structured leadership training programs or setting leadership targets appropriate to the training level; Pregnancy, childcare and work-life integration: to reduce the “taboo” associated with pregnancy in female neurosurgeons and the loss of working power both for the employer and employee. Initiatives to process pregnancy- and childcare-related career obstacles in German neurosurgery are ongoing, including the development of a DGNC-supported guideline for surgical work during pregnancy, to curtail the often months-long struggle with local authorities in which female neurosurgeons used to have almost no power or external support. Investing the effort to develop departmental and individual strategies for increasing working hours, of which the following are only examples: if medical or personal reasons hinder surgical work during pregnancy, taking on more non-surgical work may unburden colleagues, rather than being banned from the workplace, in exchange for a dedicated relaunch when returning from maternity leave with increased surgical exposure; creating scholarship programs financing temporary childcare opportunities for surgeons returning to the workplace; increasing flexibility by enabling home-office hours to which documentation can be outsourced at their own schedule; Mentoring and Networking: to create internal and external mentoring opportunities, such as by our commission. Highlighting the personas of women leaders may increase awareness that—with the same requirements for surgical and scientific excellence as for any male—this path is also possible for women. We believe there is willingness and competence of today’s women in German neurosurgery to take responsibility in leadership. The commission will engage to balance their chances and normalize their presence in the neurosurgical society.

## Study limitations

This is a primarily descriptive study on female leaders in German neurosurgery. Despite the common position, their careers proved to be highly individual, which cannot be expressed fully in this compressed summary. Due to a lack of past studies in this regard, some references were retrieved from other surgical fields, medicine or non-medical professions, or from other countries than Germany. Comparable data on male department heads but, even more so, on women who may have left the academic trajectory are hard to obtain. Whether milestones in the career paths were reached earlier or later by women versus men would require a control group of male neurosurgery department heads. The number of participating women is inherently limited and our snapshot data do not allow to deduce change over time. Thirty years lie between the first and last included female department head, limiting the comparability of social and professional conditions between careers. Long-term comparisons will need to show whether all combined efforts may translate into a more balanced gender-distribution.

## Conclusion

Female neurosurgical department chairs in Germany have assumed their positions during the last 30 years. Despite heterogeneous experiences made along their career, their achievements as leaders create visible role models for the younger, diverse generation of neurosurgeons. Pending comparisons to male department heads and long-term data are required to understand how and if gender influences career advancement and whether a change in trend is ongoing.

## Data Availability

The data and materials are available from the first author on reasonable request.
